# Benefiting from Both Ethanol Oxidation and Bidentate Thiol Groups of DHLA Ligands under Photoirradiation for Synthesis of Au Nanoparticles With Their Catalytic and Peroxidase Like Activity

**DOI:** 10.1021/acsomega.5c00274

**Published:** 2025-04-08

**Authors:** Nimet Temur, Seyma Dadi, Ayse Nur Dogan, Mustafa Nisari, Ilker Avan, Ismail Ocsoy

**Affiliations:** 1 Department of Analytical Chemistry, Faculty of Pharmacy, Erciyes University, Kayseri 38039, Turkey; 2 Department of Materials Science and Nanotechnology Engineering, Abdullah Gül University, Kayseri 38080, Turkey; 3 Department of Restorative Dentistry, Faculty of Dentistry, Erciyes University, Kayseri 38039, Turkey; 4 Department of Medical Biochemistry, Faculty of Dentistry, University of Nuh Naci Yazgan, Kayseri 38090, Turkey; 5 Department of Chemistry, Faculty of Science, Eskişehir Technical University, Eskişehir 26470, Turkey

## Abstract

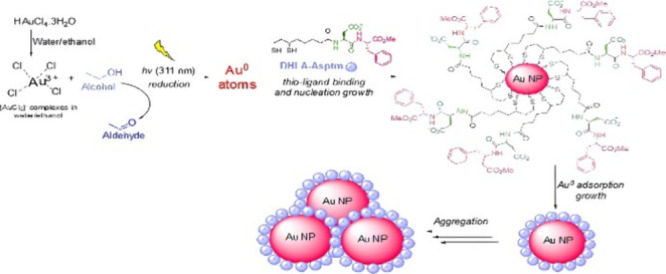

In this work, we rationally synthesized quite stable gold nanoparticles (AuNPs) using dihydrolipoic acid (DHLA) and DHLA-aspartame (DHLA-Asptm) as both reducing and stabilizing agents in a mixture of water/ethanol at RT under photoirradiation in 10 min. The novelty of this work is that benefiting from both the oxidation of ethanol to ethanal and having the bidentate thiol groups of DHLA, stable DHLA@AuNPs and DHLA-Asptm@AuNPs were successfully and rapidly formed without additional reducing reagents. We systematically examined the formation of DHLA@AuNPs and DHLA-Asptm@AuNPs under different pH values and reaction temperatures. Furthermore, the salt tolerance of DHLA@AuNPs and DHLA-Asptm@AuNPs was tested in a series of sodium chloride solutions. We showed the catalytic and peroxidase-like activities of DHLA@AuNPs against 4-nitrophenol and 3,3′,5,5′-tetramethylbenzidine. The AuNPs were characterized by UV–vis spectrophotometry, scanning transmission electron microscopy, zeta potential, and dynamic light scattering.

## Introduction

Gold nanoparticles (AuNPs) have a significant place in biomedical applications due to their unique (unrivaled) optical, chemical, and electronic properties owing to their biocompatibility, functional surface, and easy synthesis procedure.^1−8^ In addition to that, AuNPs exhibit an unrivaled surface plasmon resonance (SPR) property for many applications such as cancer therapy, optical imaging, and optical sensing.^9−14^ It is worthy to mention that the SPR property of AuNPs is affected by the size, shape, size dispersity and colloidability.^15,16^

Although various methods have been developed for the synthesis of water-soluble AuNPs,^4,16−21^ trisodium citrate-based reduction called the Turkevich method is the most used method to synthesize uniform and colloidal AuNPs.^22,23^ In principle, trisodium citrate acts as both a reductant and stabilizing agent, but boiling of gold ions (A^3+^) solution is a compulsory step for reduction of Au^3+^ to Au^0^ and formation of AuNPs. The potential drawbacks of this method are (i) the need for thermal reduction processes, (ii) easy detachment of citrate molecules from the surface of AuNPs, leading to aggregation in mild salt concentration, and (iii) potential contamination of the synthesized AuNPs owing to Na^+^ cations in the citrate molecule.^3^

On the contrary, photoreduction methods have recently received great attention for synthesis of AuNPs because light is noninvasive and controllable and has the ability to produce AuNPs in a targeted position.^24,25^

Although photoreduction methods have been actively used for the synthesis of AuNPs, they require multiple reagents for reduction and stabilization and/or need long light exposure.^26,27^ For instance, Shiraishi et al. reported the successful formation of AuNPs via exposing the benzoate–Au^3+^ complex to UV light at room temperature (RT: 25 °C).^28^ However, effective electron transfer from ligand to Au^3+^ ions for reduction and adsorption of benzoate anions on the surface of the AuNPs as stabilizing agents needs 200 min of photoirradiation. Researchers shortened the photoirradiation time until 25 min for the synthesis of AuNPs using citric acid as a reductant and surface stabilizing agent, but the easy and rapid desorption of citric acid from the surface of the AuNPs at elevated temperature and salt solution leads to irreversible AuNP aggregation, which limits the use of both this method and the AuNPs in wide application areas.^3^ Previously, the Mattoussi group combined photochemical reduction of the dithiolane group of LA with energetically favorable in situ ligand chemisorption, yielding rapid modification of the surfaces.^16^ In our previous work, we also utilized the thiol–Au chemistry for the synthesis of AuNPs using dihydrolipoic acid (DHLA) and its modified ligand in water and at RT under photoirradiation.^29^ The DHLA-directed AuNPs were formed in 10 min, however, in the presence of citric acid used as a potential reducing agent.

Herein, we rationally synthesized quite stable AuNPs using DHLA and DHLA-aspartame (DHLA-Asptm) as both reducing and stabilizing agents in a mixture of water/ethanol at RT under photoirradiation with a wavelength of 311 nm within 10 min. Benefiting from both the oxidation of ethanol to ethanal and the bidentate thiol groups of DHLA, stable DHLA@AuNPs and DHLA-Asptm@AuNPs were successfully and rapidly formed without any reducing reagents. We systematically examine the formation of DHLA@AuNPs and DHLA-Asptm@AuNPs under different pH values and reaction temperatures. Furthermore, the salt tolerance of both DHLA@AuNPs and DHLA-Asptm@AuNPs were tested in a series of sodium chloride (NaCl) solutions. We showed the catalytic and peroxidase-like activities of DHLA@AuNPs against 4-nitrophenol (4-NP) and 3,3′,5,5′-tetramethylbenzidine (TMB).

## Experimental Methods

### Materials and Instrumentation

Gold(III) chloride trihydrate, 4-NP, NaCl, hydrogen peroxide (H_2_O_2_), TMB, and lipoic acid were purchased from Sigma-Aldrich and used as received. A UV fluorescent narrowband lamp (Philips PL-S 2 × 9 W) with a wavelength of 311 nm was used in the photoreductive synthesis of AuNPs. We used a UV–vis spectrometer for absorbance measurement, scanning transmission electron microscopy (STEM) for AuNP morphology studies, zeta potential (ZT) for surface charge analysis, and dynamic light scattering (DLS) for AuNP hydrodynamic size determination. Dihydrolipoic acid **2** and lipoyl benzotriazole **3** were prepared according to a previously reported procedure.^30^

### Synthesis of the Capping Ligand Dihydrolipoyl Aspartame (DHLA-Asptm)

**Figure 1:**
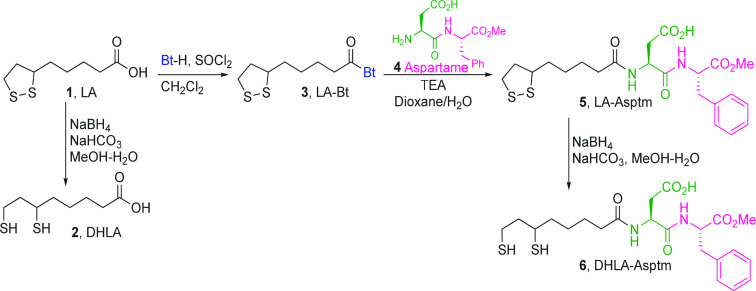
Synthesis of the capping ligands **2** (DHLA) and **6** (DHLA-Asptm).

Triethylamine (15 mmol) was added to a suspension of dipeptide **4** (Asptm) (15 mmol) in water (6 mL) at 10 °C (Figure 1). A solution of lipoyl benzotriazole **3** (10 mmol) in 15 mL of 1,4 dioxane was added dropwise to this solution, and the mixture was stirred for 6 h at RT. The progress of the reaction was monitored with TLC using EtOAc/hexane (1:2) solution. After completion of the reaction, the excess solvent was removed, and 40 mL of water was added to the remaining suspension. The solution was then acidified to a pH of 2–3 by adding 4 N HCl. The resulting solution was extracted with CH_2_Cl_2_ (3 × 20 mL). The collected organic phase was washed with 4 N HCl (3 × 10 mL) and saturated NaCl solution (10 mL) and then dried over Na_2_SO_4_. The excess solvent was removed under reduced pressure, yielding white microcrystals as a mixture of diastereomers of lipoylaspartame (5, LA-Asptm, 85%), mp: 92–94 °C. ^1^H NMR (400 MHz, CDCl_3_) δ 7.32–7.14 (m, 5H), 7.10 (d, *J* = 6.6 Hz, 1H), 6.82 (d, *J* = 7.9 Hz, 1H), 4.88–4.72 (m, 2H), 3.70 (s, 3H), 3.60–3.50 (m, 1H), 3.24–2.96 (m, 4H), 2.90 (dd, *J* = 17.0, 4.5 Hz, ^1^H), 2.67 (dd, *J* = 17.1, 6.5 Hz, ^1^H), 2.50–2.36 (m, 1H), 2.24–2.10 (m, 2H), 1.94–1.84 (m, 1H), 1.74–1.30 (m, 6H). ^13^C NMR (100 MHz, CDCl3) δ 174.73, 173.51, 171.67, 170.49, 135.79, 129.37, 128.72, 127.33, 56.45, 56.42, 53.79, 52.68, 49.07, 40.38, 40.36, 38.63, 37.74, 36.21, 36.18, 35.84, 34.71, 28.90, 25.24; HRMS: *m*/*z* [M + H]^+^ calc. for C_22_H_31_N_2_O_6_S^2+^: 483.1618; found: 483.1633; [M + Na]^+^ calc. for: C_22_H_30_N_2_NaOS^2+^: 505.1437; found: 505.1454. ^1^H NMR, ^13^C NMR, COSY and HSQC NMR spectra of **5** (LA-Asptm) were shown in Figure S1–4.

### Reduction of Lipoyldipeptide

The lipoyldipeptide, **5** (LA-Asptm, 1 mmol), was added to the solution of 10 mL of aqueous methanol and 5 mL of 0.25 M NaHCO_3_. Then, 4 mmol of sodium borohydride (NaBH_4_) was slowly added to this mixture at 0 °C in an ice bath. The mixture was stirred for 1 h at 10 °C. The volatile part of the solution was removed under reduced pressure. The pH of the solution was adjusted to 2–3 by adding 4 N HCl. The solution was then extracted with EtOAc (3 × 10 mL). The organic phase was washed with 10 mL of saturated NaCl solution and dried over Na_2_SO_4_. The solvent was removed under reduced pressure, resulting in a hygroscopic solid of dihydrolipoylaspartam (**6**, DHLA-Asptm, 95%). White microcrystals as a mixture of diastereomers, mp: 126 – 127 °C. ^1^H NMR (400 MHz, CDCl3) δ 7.32 – 7.18 (m, 4H), 7.14 – 7.09 (m, 2H), 6.91 (d, J = 8.0 Hz, 1H), 4.96 – 4.84 (m, 1H), 4.82 – 4.74 (m, 1H), 3.70 (s, 3H), 3.14 (dd, J = 14.0, 5.7 Hz, 1H), 3.03 (dd, J = 13.9, 6.8 Hz, 1H), 2.95 – 2.84 (m, 2H), 2.79 – 2.56 (m, 3H), 2.25 – 2.13 (m, 2H), 1.96 – 1.82 (m, 1H), 1.80 – 1.68 (m, 1H), 1.65 – 1.41 (m, H), 1.30 (dd, J = 7.7, 2.7 Hz, 1H). ^13^C NMR (100 MHz, CDCl_3_) δ 174.70, 173.68, 171.68, 170.55, 135.80, 129.36, 128.71, 127.30, 53.82, 52.67, 49.12, 42.88, 39.45, 39.43, 38.83, 37.73, 36.22, 35.96, 26.67, 25.16, 22.44; HRMS: *m*/*z* [M + H]^+^ calc. for C_22_H_33_N_2_O_6_S^2+^: 485.1775; found: 485.1788; [M + Na]^+^ calc. for: C_22_H_32_N_2_NaO_6_S^2+^: 507.1594; found: 507.1610. ^1^H NMR, ^13^C NMR, COSY, and HSQC NMR spectra of **6** (DHLA-Asptm) are shown in Figures S5–S8.

### Synthesis and Characterization of Gold Nanoparticles (AuNPs)

**Figure 2:**
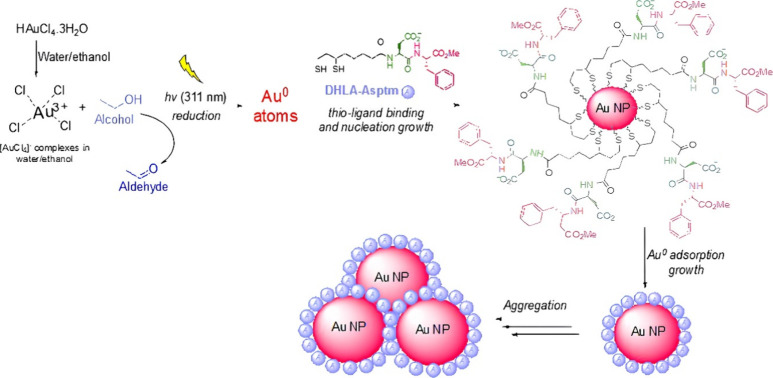
Potential mechanism for the synthesis of DHLA-Asptm@AuNPs.

First, 0.5 mM Au^3+^ in water and 0.5 mM DHLA and DHLA-Asptm solutions in EtOH were prepared. In a glass vial, 1 mL of 0.5 mM Au^3+^ solution was separately mixed with 0.25 mL of DHLA and DHLA-Asptm solutions. Each mixture was exposed to UV radiation (Figure 2). The solution turned from pale-yellow to red purple after 10 and 20 min of UV exposure, which indicates formation of DHLA@AuNPs and DHLA-Asptm@AuNPs. The AuNPs were centrifuged at 12,000 rpm for 15 min to remove unreacted excess ligands and Au^3+^ ions. The AuNPs were then stored at 4 °C for characterization and activity tests. The characteristic localized surface plasmon resonance (LSPR) absorbance peaks of AuNPs in water were recorded at around 525 nm by a UV–vis spectrophotometer. Images of the AuNPs were produced by STEM. The surface charge and effective diameter of the AuNPs were determined by using ZT and DLS methods, respectively.

It is already well documented that there is no linear decrease or increase in precursor, reducing, and even stabilizing agent concentrations in the synthesis of NPs. This means that when the concentrations of Au^3+^ ions and reducing agents are increased or decreased, the formation of NPs cannot be guaranteed. For instance, we used Au^3+^ ions with 2 and 10 mM concentrations for synthesis of DHLA-directed AuNPs as shown in Figure S9.

### Catalytic and Enzyme-like Activities of AuNPs

Previous reports showed the reduction of 4-NP to 4-AP. Two mL of 0.2 mM 4-NP solution was prepared, and 0.045 and 0.030 M concentrations of sodium borohydride (NaBH_4_) were mixed at RT. Then, 0.5 mL of 5 nM DHLA@AuNPs and DHLA-Asptm@AuNPs solutions were immediately added to each mixture. Finally, the reduction of 4-NP to 4-AP was recorded by a UV–vis spectrometer.

The enzyme-like activity of the AuNPs was determined through oxidation of the TMB substrate to a blue-colored product in the presence of hydrogen peroxide (H_2_O_2_).^31,32^ The enzymatic activity observed depends on TMB, H_2_O_2_, and AuNP concentration.

## Results and Discussion

The mechanism for the photochemical conversion of Au^3+^ ions to AuNPs has been thoroughly investigated in both aqueous^33−37^ and alcoholic solvents.^26,38−42^ Photoirradiation for the ligand-to-metal charge transfer (LMCT) bands of the AuCl_4_^–^ complex in alcohol–water solution enables the dissociation of Au–Cl bonds and the reduction of Au^3+^ to Au^0^ particles through successive reduction steps (Au^3+^→ Au^2+^ → Au^1+^) while simultaneously oxidizing the alcohol.^26^ Photoexcitation of the DHLA-[AuCl_4_]^−^ initiates the nucleation step by producing the formation of Au^0^ seeds through the oxidation of ethanol. Then, coalescence of Au^0^ seeds leads to growth of AuNPs and eventual AuNP formation by DHLA and DHLA-Asptm bound onto the surface of the AuNPs acting as stabilizing agents as given in eq 1.
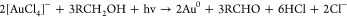
1

The reaction rate and mechanism, including intermediates, byproduct, and product sizes, can vary depending on the type of gold (Au) salt, solution pH, the reducing agents used, and the presence of additives such as alcohols and polymers.^33,36,43^ The Au^3+^ reduction reaction rate is significantly influenced by the pH of the solution in both wet and photochemical processes because it affects the speciation of the [AuCl_4_]^−^ complex.^23,33−36,44^ In wet chemical reactions, as the pH increases, chloride (Cl^–^) ligands are exchanged for hydroxide (OH) ligands, resulting in the formation of less reactive complexes such as [AuCl_2_(OH)_2_]^−^ and [AuCl(OH)_3_]^−^, and the initial reaction rate was reduced.^23^ On the other hand, when the reaction is carried out in water under strong field laser irradiation, a higher reduction rate under basic conditions is observed due to the increasing number of reactive reducing species on water photolysis, which overcomes the lower reactivity of [Au(OH)_4_]^−^ as compared to [AuCl_4_]^−^.^33^ Additionally, it has been reported that the photoinduced formation of AuNPs is inhibited in the presence of thiol compounds.^45^ When the concentration of biothiols exceeds that of Au(III), the formation of AuNPs is almost completely suppressed due to the creation of Au(III)–biothiol complexes.

Aspartame (Asptm), an artificial sweetener, is a dipeptide made up of aspartic acid and the methyl ester form of phenylalanine. As a dipeptide figure, it can inherently interact with biological systems.^46^ Because of its biocompatibility, cost-effectiveness, and ease of replacement with other biomolecules, Asptm may serve as an ideal material for surface coatings on nanoparticles.^47,48^ In this regard, gold–silver core–shell nanostructures stabilized with Asptm have shown remarkable stability in aqueous environments and a prolonged shelf life.^47^ Additionally, under physiological conditions, Asptm can spontaneously self-assemble, leading to the formation of regular β-sheet-rich nanofibrils with amyloid characteristics. This process occurs through hydrogen bonds and π–π interactions.^49^

In this study, we present the in situ photoinduced synthesis of ultrastable AuNPs using a bidentate biothiol group of DHLA, a reduced form of apha-lipoic acid (LA), and DHLA-Asptm in a water–ethanol mixture taking advantage of the oxidation of ethanol to acetaldehyde. When AuNPs are formed under UV irradiation, both DHLA and DHLA-Asptm ligands help stabilize the AuNPs by binding to them with their bidentate thiol groups. This interaction increases the dispersion of the nanoparticles in aqueous solutions, aided by the hydrogen bonding capabilities of the carboxylic and peptide moieties present in the ligands. The concentration of biothiols was taken to less than half that of the Au(III) concentration to avoid inhibiting the formation of AuNPs.

### Synthesis and Characterization of DHLA@AuNPs and DHLA-Aspmt@AuNPs

In the synthesis of DHLA@AuNPs and DHLA-Aspmt@AuNPs, 1 mL of 0.5 mM HAuCl_4_ solution and 0.25 mL of 0.5 mM each ligand was added in a glass vial one by one and exposed to UV light at pH ≈ 2.5 for time-dependent periods. The potential reaction between thiol and Au^3+^ to form the AuNP structure is given step by step in Figure 2. Briefly, both DHLA@AuNPs and DHLA-Aspmt@AuNPs NPs are formed in three sequential stages in which (1) the Au^3+^ is first sequentially reduced to Au^1+^ over Au^2+^ via the oxidation of ethanol to acetaldehyde, (2) while an early Au^0^ is formed from Au^1+^ by again solvent oxidation, the nucleation, autocatalytic growth, and aggregative growth processes concurrently proceed, and (3) last, AuNPs appear after an aggregative growth.

The first DHLA-capped AuNPs were prepared by Roux et al.,^50^ applying Brust’s protocol to improve the colloidal stability of NPs. And lately, within the same protocol, many functionalized AuNPs via capping of LA and DHLA ligands have been used as anticancer,^51^ antimicrobial,^52^ and nanosensor^53^ agents. In our studies, we preferred the use of DHLA coupled derivatives over LA couples because of their better chelating ability with two −SH units and to avoid the formation of the decomposed products related to LA under UV irradiation.

Our photoreduction studies are conducted using narrow-band UV light (2 × Philips 9W TL01, max 311 nm, PLS type), which perfectly fits the range of absorbance wavelengths (around 320 nm) of AuCl_4_^–^ ions to become excited. As it is well-known that the excited molecules undergo reduction or oxidation much easier than in their ground states,^42^ the use of narrow-band UV light with 311 nm would simplify the reducing process of AuCl_4_^–^. Also, the irradiation band of this lamp pretty much covers the absorption band of the LA and DHLA derivatives.

**Figure 3:**
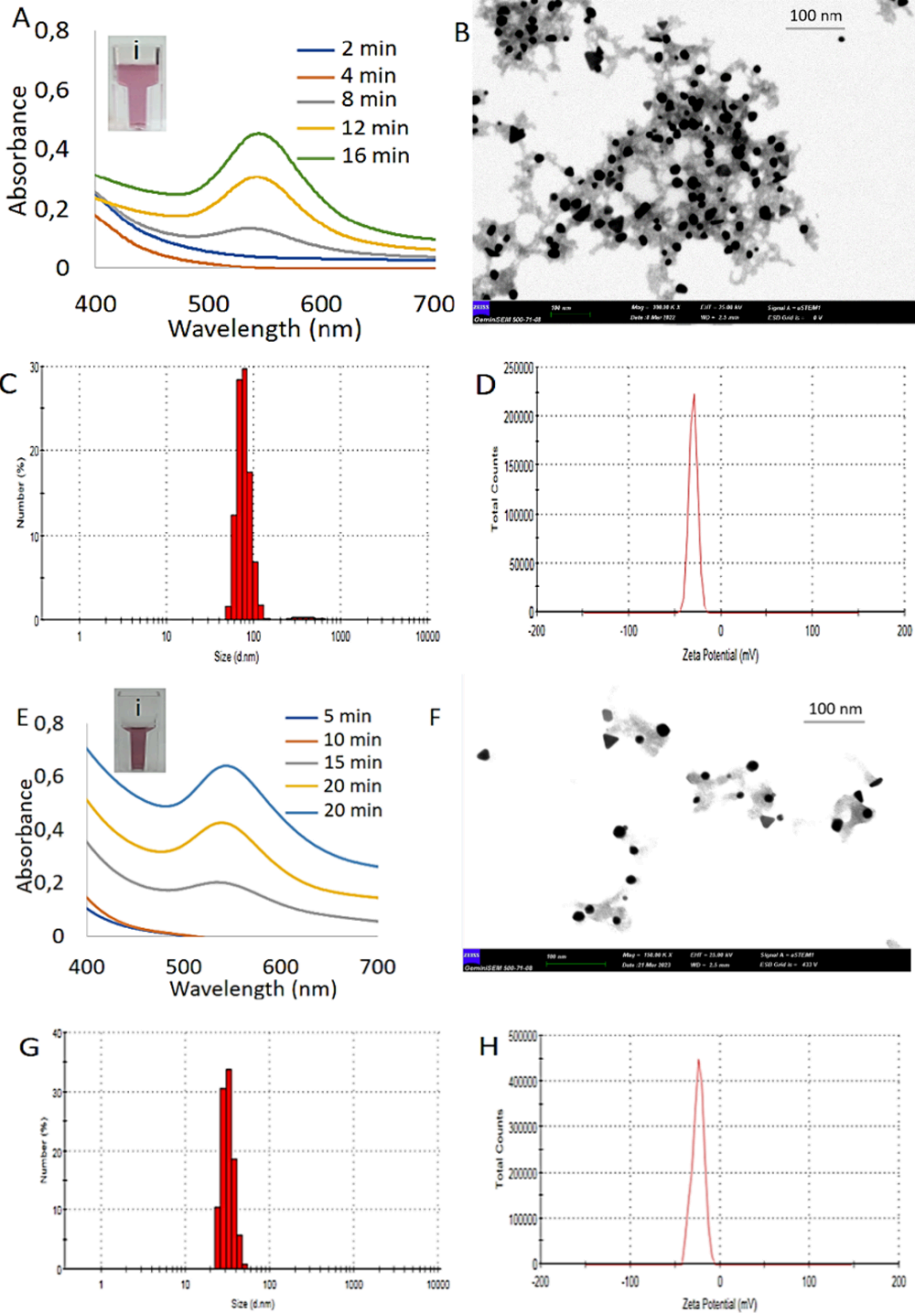
Characterization of the AuNPs. (A) Time-dependent UV–vis spectra, (B) STEM image, (C) DLS spectrum, and (D) zeta potential of DHLA@AuNPs. (E) Time-dependent UV–vis spectra, (F) STEM image, (G) DLS spectrum, and (H) zeta potential of DHLA-Asptm@AuNPs.

Formation of DHLA@AuNPs and DHLA-Aspmt@AuNPs was characterized with various techniques as shown in Figure 3. Figure 3A gives the first characteristic LSPR absorption peak of DHLA@AuNPs formed in 8 min of UV irradiation at around 535 nm (gray line). More intense and distinct peaks for DHLA@AuNPs formed in 12 min (yellow line) and 16 min (green line) of UV irradiation were recorded around 535 nm. We claim that peak intensity is proportional to the number of the formed AuNP or AuNP concentration. The reddish color of DHLA@AuNPs can be seen in Figure 3A (i). The STEM image showed that the DHLA@AuNPs are spherical and quite uniform with a diameter of 17 nm (Figure 3B). The hydrodynamic size and surface charge of the DHLA@AuNPs were determined to be 80 nm (PDI: 0.313) and −30.1 mV in Figure 3C,D, respectively. Similarly, characteristic LSPR absorption peaks of DHLA-Aspmt@AuNPs and solution color are very consistent with those of DHLA@AuNPs (Figure 3E (i)). However, DHLA-Aspmt@AuNPs are somehow small around 13 nm compared to DHLA@AuNPs as seen in the STEM image (Figure 3F). The hydrodynamic size and surface charge of the DHLA-Aspmt@AuNPs were measured around 65 nm (PDI: 0.632) (Figure 3G) and −24.4 mV (Figure 3H).

### Effect of pH on the Formation of the AuNPs

**Figure 4:**
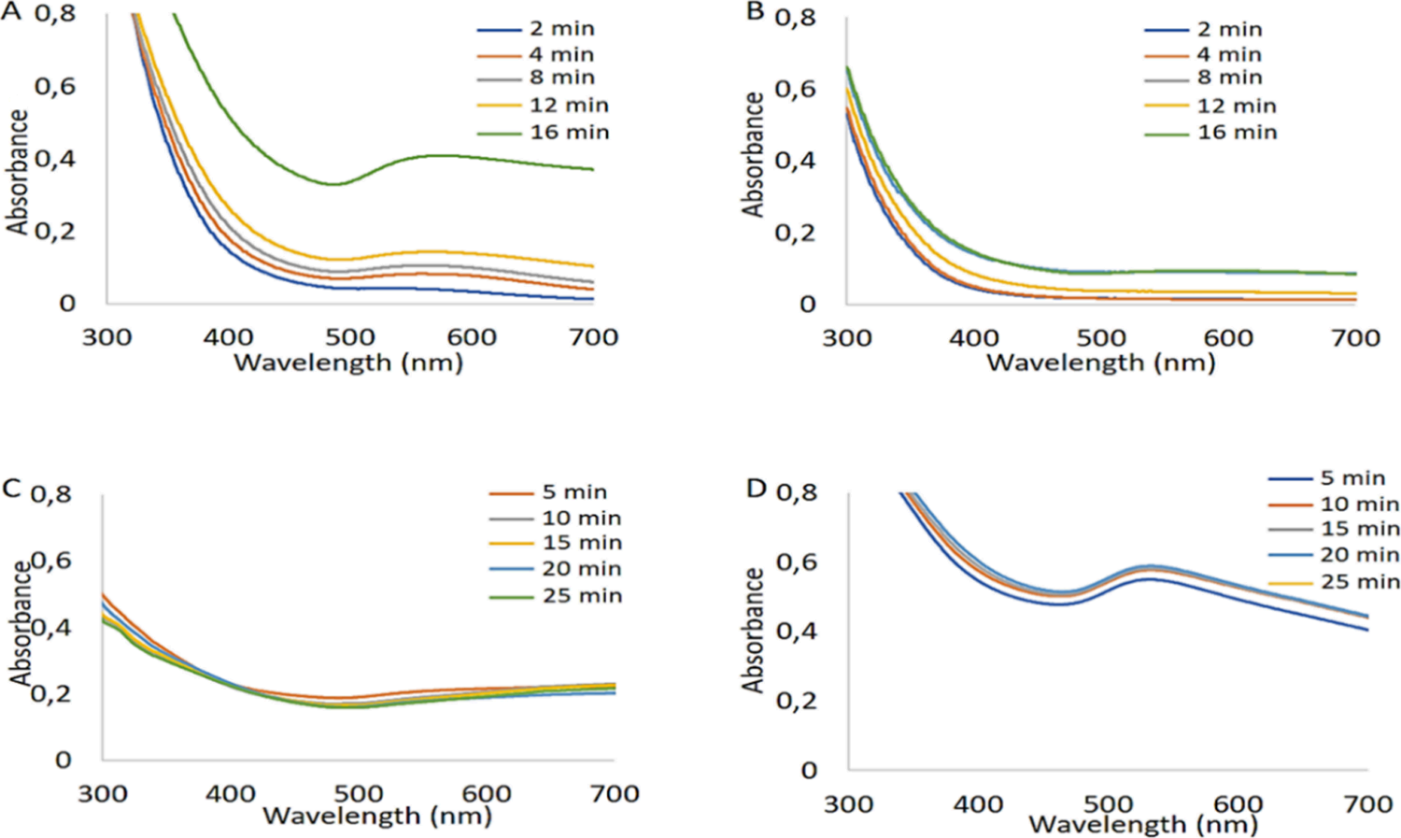
Time-dependent formation of the AuNPs at pH 7.4 and 11.5. Absorbance spectra of DHLA@AuNPs synthesized at (A) pH 7.4 and (B) pH 11.5. Absorbance spectra of DHLA-Asptm@AuNPs synthesized at (C) pH 7.4 and (D) pH 11.5.

For further study, the effect of pH on the formation of the AuNPs was examined. Ligand–Au^3+^ solutions were prepared at different pHs using NaOH. The speciation of aqueous [AuCl_4_]^−^ is largely influenced by pH, which also affects the size and properties of the forming AuNPs.^44^ At low pH levels, the predominant species of aqueous HAuCl_4_ consists of [AuCl_*x*_(OH)_4–*x*_]^−^ (where *x* ≥ 2). In contrast, at higher pH levels, the species shifts to [AuCl_*x*_(OH)_4–*x*_]^−^ (where *x* < 2).^43^ In our study, all species of [AuCl_*x*_(OH)_4–*x*_]^−^, which depend on pH, also compete with the thiol groups of DHLA, which have previously shown an inhibitory effect on AuNP formation. Figure 4 shows that the proper absorbance peaks for DHLA@AuNPs and DHLA-Aspmt@AuNPs were observed at both pH 7.4 and 11.5 since these AuNPs gave strong absorbance peaks in the original synthesis pH (pH ≈ 2.5). The species [AuCl(OH)]^−^ predominantly occurs at a pH of 7.4, while [Au(OH)_4_]^−^ is mostly present at a pH of 11.5. Both of these species are less reactive compared to [AuCl_3_(OH)]^−^, which predominates at a pH of 2.5 in the reduction of Au(III).^23^ Providing this information, the DHLA@AuNPs were formed by 16 min of photoirradiation at pH 7.4 and gave very broad absorbance peaks (green line in Figure 4A) in 565–575 nm while the DHLA@AuNPs were formed by 8 min of photoirradiation at pH 2.5. Nevertheless, no absorbance peaks for these AuNPs were seen at pH 11.5 (Figure 4B), as might be the indication of no AuNP formation due to the inhibition effect of thiol groups on DHLA. Interestingly, when DHLA-Asptm, a dipeptide analogue of DHLA, was used at pH 7.4, no formation of AuNPs was observed during any photoirradiation time (Figure 4C). Additionally, quite acceptable absorbance peaks for DHLA-Asptm@AuNPs synthesized even by 5 min of photoirradiation at pH 11.5 appeared around 535 nm, which is probably due to the breakdown of the thio–Au complex at this pH (Figure 4D).

**Figure 5:**
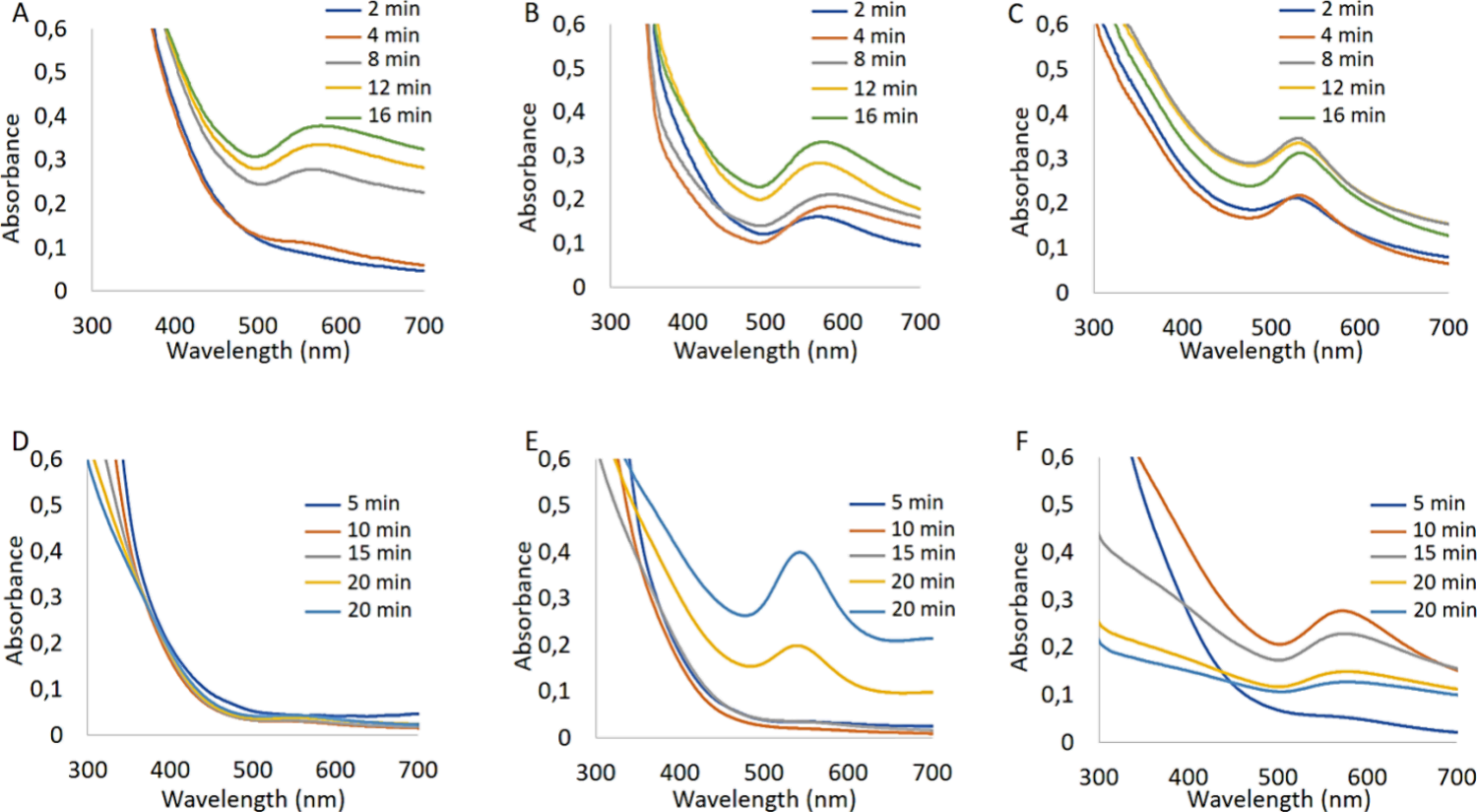
Effect of the reaction temperature for the observation of AuNP formation with UV spectra. Absorbance spectra of DHLA@AuNPs synthesized at (A) at 40 °C, (B) at 60 °C, and (C) at 80 °C. Absorbance spectra of DHLA-Asptm@AuNPs synthesized at (D) at 40 °C, (E) at 60 °C, and (F) at 80 °C.

We also examined how the reaction temperature influences the formation of the AuNPs under photoirradiation in Figure 5. For instance, while DHLA@AuNPs synthesized at 40 °C gave broad absorbance peaks around 575 nm after 8 min of photoirradiation, DHLA@AuNPs synthesized by 2 min of photoirradiation at 60 °C showed absorbance peaks at the same wavelength (575 nm) as presented in Figure 5A,B, respectively. It seems that DHLA@AuNPs were formed much faster at 60 °C than at 40 °C. We claim that an increase in reaction temperature expedites electron transfer from ligand to Au^3+^ ions and leads to rapid AuNP formation. This claim was supported by the DHLA@AuNPs synthesized at 80 °C as shown in Figure 5C. DHLA@AuNPs synthesized by any photoirradiation time including 2 min at 80 °C gave a quite narrow and kind of sharp absorbance peak at around 535 nm. In contrast to that, there were no absorbance peaks observed for DHLA-Asptm@AuNPs synthesized at 40 °C (Figure 5D). This means that no AuNPs were formed at 40 °C. DHLA-Asptm@AuNPs synthesized at 60 °C gave first and narrow absorbance peaks around 545 nm after 12 min of photoirradiation (Figure 5E). The DHLA-Asptm@AuNPs were synthesized at 80 °C under various periods of photoirradiation; interestingly, the absorbance peaks were recorded around 580 nm after only 4 and 8 min of photoirradiation (Figure 5F). Although elevated temperature resulted in rapid AuNP formation, an almost 35 nm right shift and broad peaks on the absorbance spectrum were observed, which indicates the aggregation of the AuNPs.

**Figure 6:**
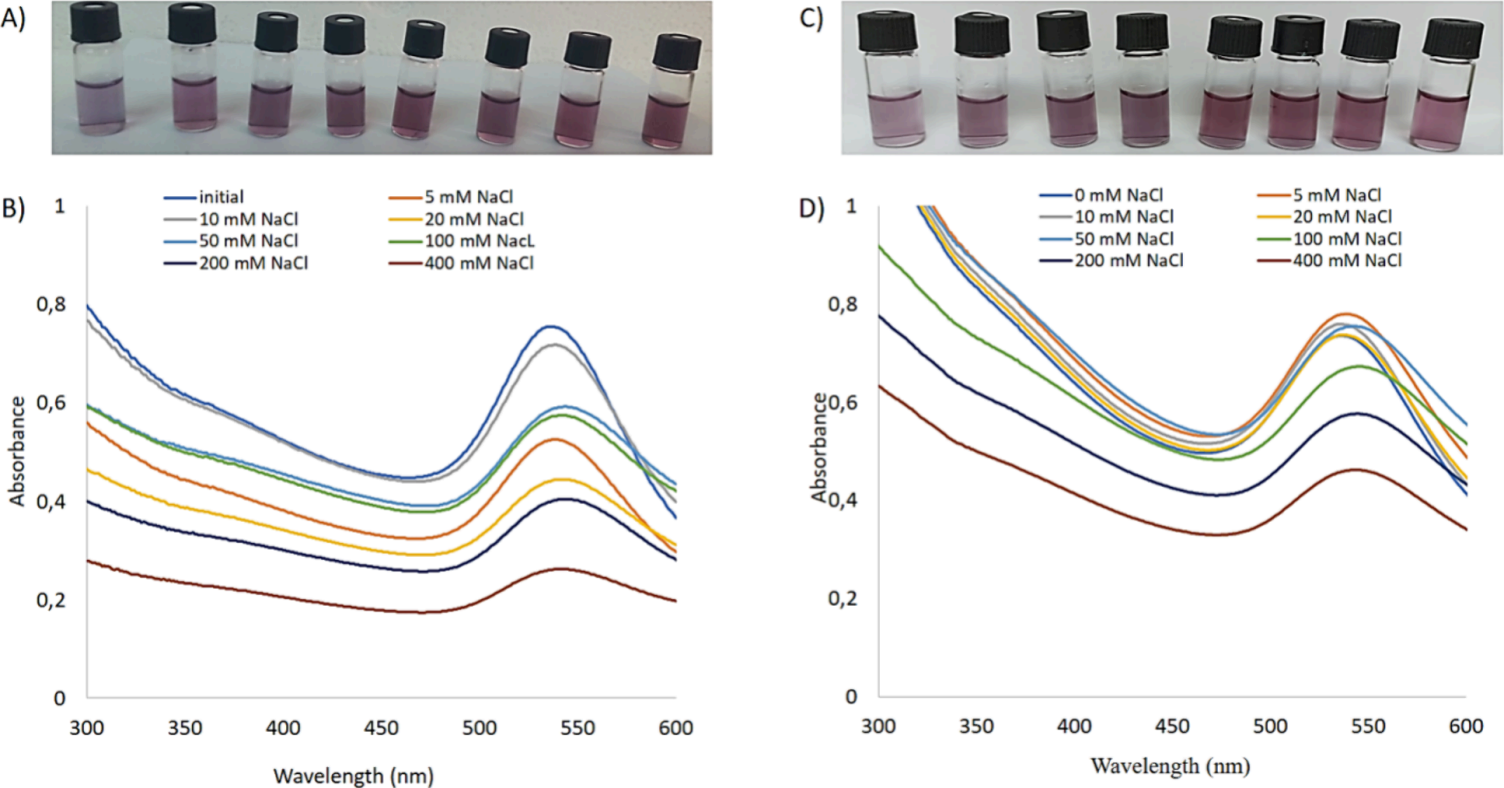
Salt tolerance of the AuNPs. (A) Photos of DHLA@AuNPs dispersed in various concentrations of NaCl solutions. (B) UV–vis spectra of the photos of DHLA@AuNPs dispersed in various concentrations of NaCl solutions. (C) Photos of DHLA-Asptm@AuNPs dispersed in various concentrations of NaCl solutions. (D) UV–vis spectra of DHLA-Asptm@AuNPs dispersed in various concentrations of NaCl solutions.

Almost all NPs exhibit efficient physicochemical and biological properties as long as they are colloidal.^29,54−57^ It is well-known that AuNPs have been actively used in various biomedical applications so-called “gold medicine” owing their stability in different experimental conditions.^58−61^ However, when AuNPs aggregate, they may lose their unique physicochemical and biological properties. As a systematic study, we tested the salt tolerance capability of DHLA@AuNPs and DHLA-Asptm@AuNPs, and results are shown in Figure 6. The synthesized AuNPs by the Turkevich methods was not stable in over 50 nM NaCl solution. They rapidly formed aggregation and the original wine-red color of the AuNPs turned into blue color (data not shown here) due to detachment of weakly bound citric acid from the AuNP surface. It is worthy to mention that both DHLA@AuNPs and DHLA-Asptm@AuNPs were dispersed in 400 mM NaCl solution, but no remarkable color change was seen in their original reddish color as shown in Figure 6A,C, respectively. The stability of DHLA@AuNPs and DHLA-Asptm@AuNPs in highly concentrated NaCl solutions was confirmed with absorbance spectra. While the initial absorbance peaks of both DHLA@AuNPs and DHLA-Asptm@AuNPs were recorded at 525 nm, there was no shift observed on their absorbance spectra as given in Figure 6B,D. We interpret that both DHLA and DHLA-Asptm ligands strongly bind to the surface of AuNPs owing to thiol–gold chemistry and their bidentate thiol groups.

### Catalytic and Enzymatic Activities of the Synthesized AuNPs

**Figure 7:**
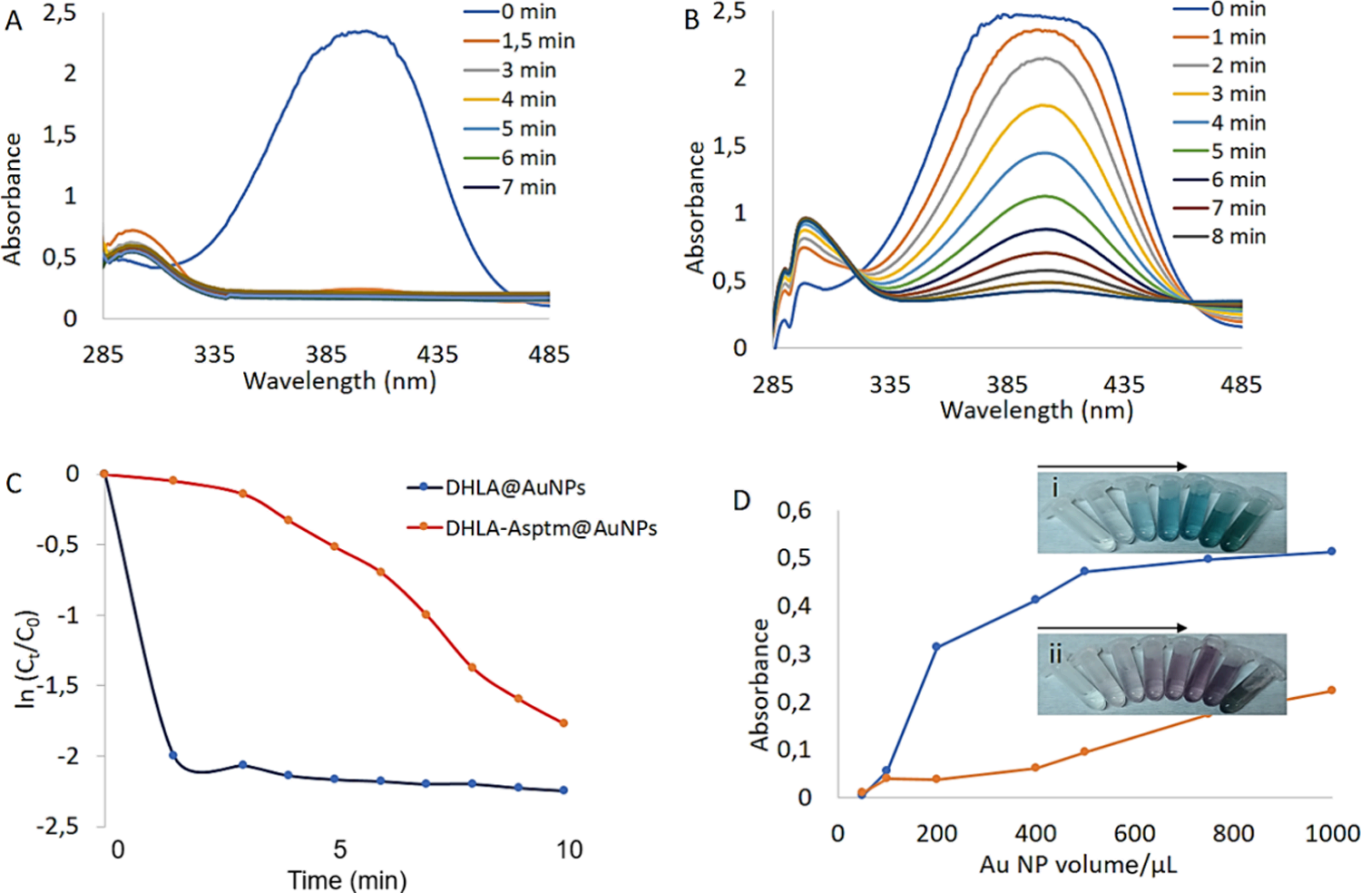
Conversion of 4-NP to 4-AP catalyzed by (A) DHLA@AuNPs and (B) DHLA-Asptm@AuNPs. (C) Plots of −*kt* = ln(*C_t_*/*C*_0_) of 4-NP and (D) peroxidase-like activity of the AuNPs against TMB.

In general, various metallic NPs such as gold and silver NPs act as novel catalysts with great performance.^62−65^ Herein, conventional catalytic experiments were carried out with the AuNPs. Both DHLA@AuNPs and DHLA-Asptm@AuNPs catalyzed the reduction of 4-nitrophenol (4-NP) to 4-aminophenol (4-AP) in the presence of NaBH_4_ used as a hydrogen source in aqueous solution as seen in Figure 7A,B, respectively. However, DHLA@AuNPs caused much faster conversion of 4-NP to 4-AP than did DHLA-Asptm@AuNPs. These results were well consistent with Figure 7C, showing the reaction kinetic plot. *k* is the rate constant, *C*_0_ is the initial concentration of 4-NP, and *C_t_* is the concentration of 4-NP at time *t*. The plots of −*kt* = ln(*C_t_*/*C*_0_) of both AuNP catalysts are shown. During the catalytic reaction, while the characteristic absorbance peaks of 4-NP molecules at 400 nm were decreased, the increase in the absorbance peaks of 4-AP that appeared at 300 nm was observed in the presence of both AuNPs.

AuNPs can also exhibit peroxidase-like activities in the presence of hydrogen peroxide against various targets.^66−70^ DHLA@AuNPs possess efficient peroxidase-like activity by catalyzing the oxidation of the substrate TMB in the presence of H_2_O_2_ to produce oxidized-TMB (TMBox) with blue color in aqueous solution (Figure 7D).

## Conclusions

We developed a new and rapid synthesis of ultrastable colloidal uniform AuNPs with newly designed ligands. The specific sulfur groups, which are components in DHLA and DHLA-Asptm, acted as reducing and stabilizing agents for Au ions. Only after 10 min of UV light irradiation of HAuCl_4_ and the ligands that uniform and stable AuNPs were formed. UV irradiation and ligands provided the spherical shape of AuNPs under room conditions. The AuNPs exhibited high enhanced colloidal stability compared with citrate-capped AuNPs in concentrated NaCl solution. In addition, DHLA@Au and DHLA-Asptm@AuNPs exhibited efficient catalytic activity for reduction of 4-NP to 4-AP. Finally, we observed peroxidase-like activity of the AuNPs, which showed a colorimetric change when oxidizing TMB in the presence of H_2_O_2_. We believe that functional AuNPs synthesized with DHLA and DHLA-Asptm ligands as stabilizer agents will be applied for rapid, cheap, and colorimetric nanosensors in biomedical applications.

## Supporting Information Available

^1^H NMR spectra of LA-Asptm, ^13^C NMR spectra of LA-Asptm, COSY NMR spectra of LA-Asptm, HSQC NMR spectra of LA-Asptm, ^1^H NMR spectra of DHLA-Asptm, ^13^C NMR spectra of DHLA-Asptm, COSY NMR spectra of DHLA-Asptm, and HSQC NMR spectra of DHLA-Asptm (PDF)
