# Supplementary material for: Benefiting from Both Ethanol Oxidation and Bidentate Thiol Groups of DHLA Ligands under Photoirradiation for Synthesis of Au Nanoparticles With Their Catalytic and Peroxidase Like Activity
Source: ACS Omega. 2025 Apr 8;10(15):15484–92. doi: 10.1021/acsomega.5c00274 (PMC12019526; doi:10.1021/acsomega.5c00274)
Supplement: Supplementary file 1 — ao5c00274_si_001.pdf [file ao5c00274_si_001.pdf]

SUPPORTING INFORMATION

Benefiting from Both Ethanol Oxidation and Bidentate Thiol Groups of DHLA Ligands Under Photoirradiation for Synthesis of Au Nanoparticles with Their Catalytic and Peroxidase Like Activity

Nimet Temur[1], Seyma Dadi[2], Ayse Nur Dogan[3], Mustafa Nisari[4], Ilker Avan[5,*], Ismail Ocsoy[1,*]

[1]Department of Analytical Chemistry, Faculty of Pharmacy, Erciyes University, Kayseri 38039, Turkey

[2]Department of Nanotechnology Engineering, Abdullah Gül University, Kayseri 38080, Turkey

[3]Department of Restorative Dentistry, Faculty of Dentistry, Erciyes University, Kayseri, Turkey

[4]Department of Medical Biochemistry, Faculty of Dentistry, University of Nuh Naci Yazgan, Kayseri 38090, Turkey

[5]Department of Chemistry, Faculty of Science, Eskişehir Technical University, Eskişehir 26470, Turkey

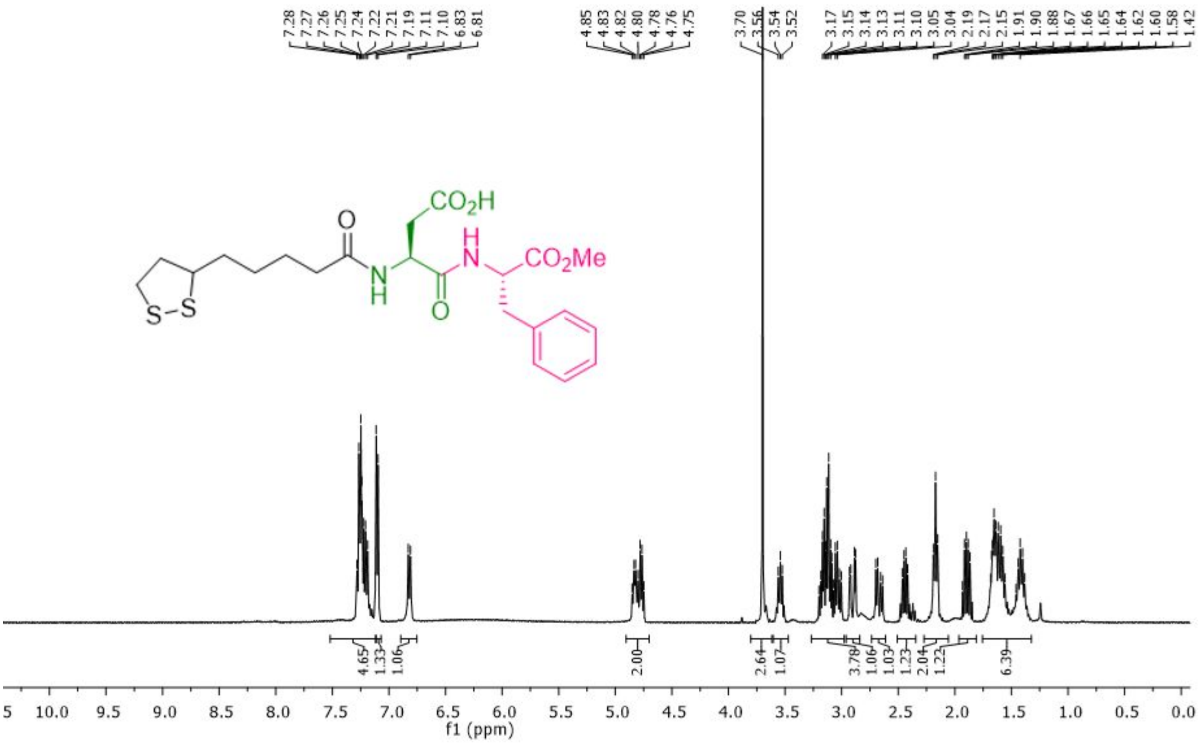

Figure S1 ^{1}H NMR spectra of **LA-Asptm**

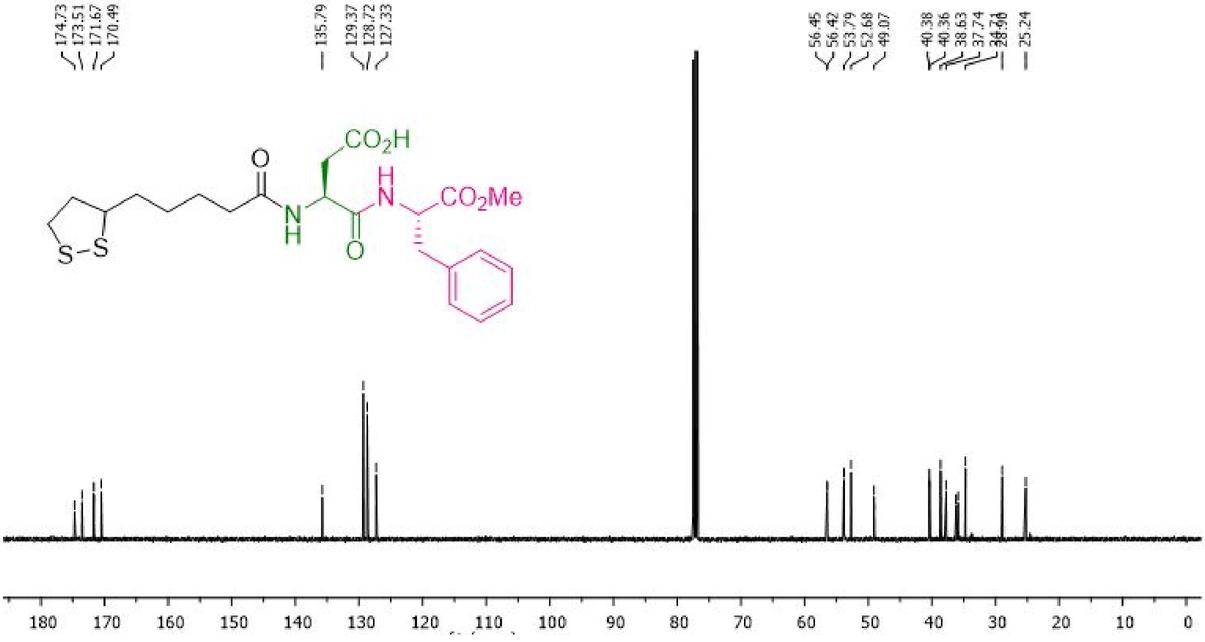

Figure S2 ^{13}C NMR spectra of **LA-Asptm**

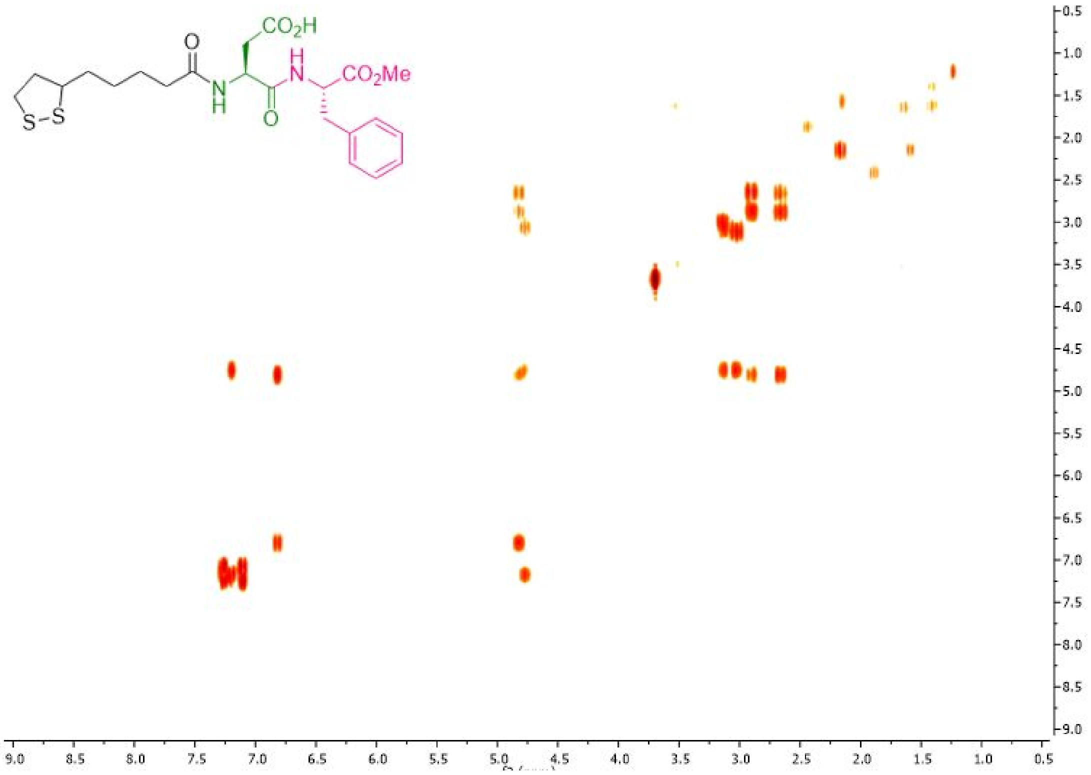

Figure S3 COSY NMR spectra of **LA-Asptm**

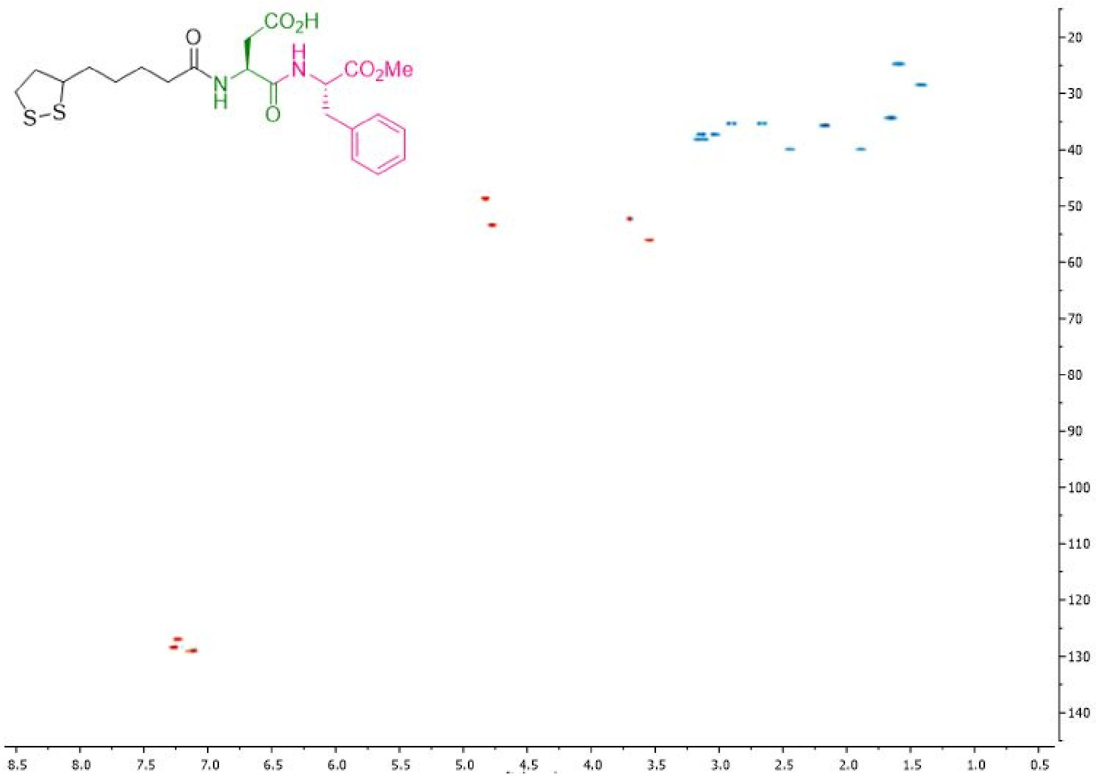

Figure S4 HSQC NMR spectra of **LA-Asptm**

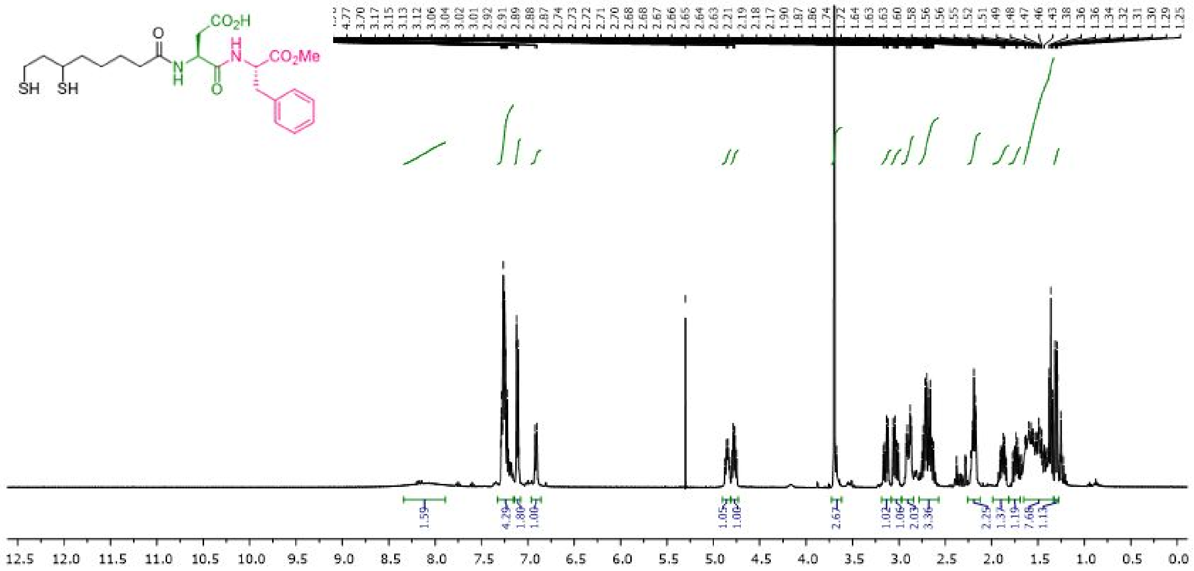

Figure S5 ^{1}H NMR spectra of **DHLA-Asptm**

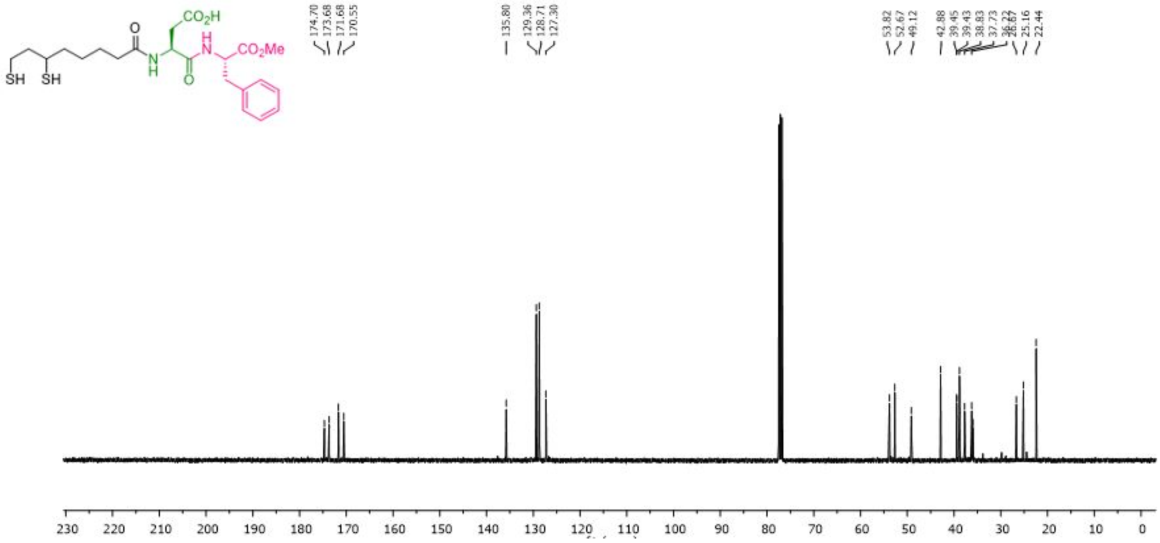

Figure S6 ^{13}C NMR spectra of **DHLA-Asptm**

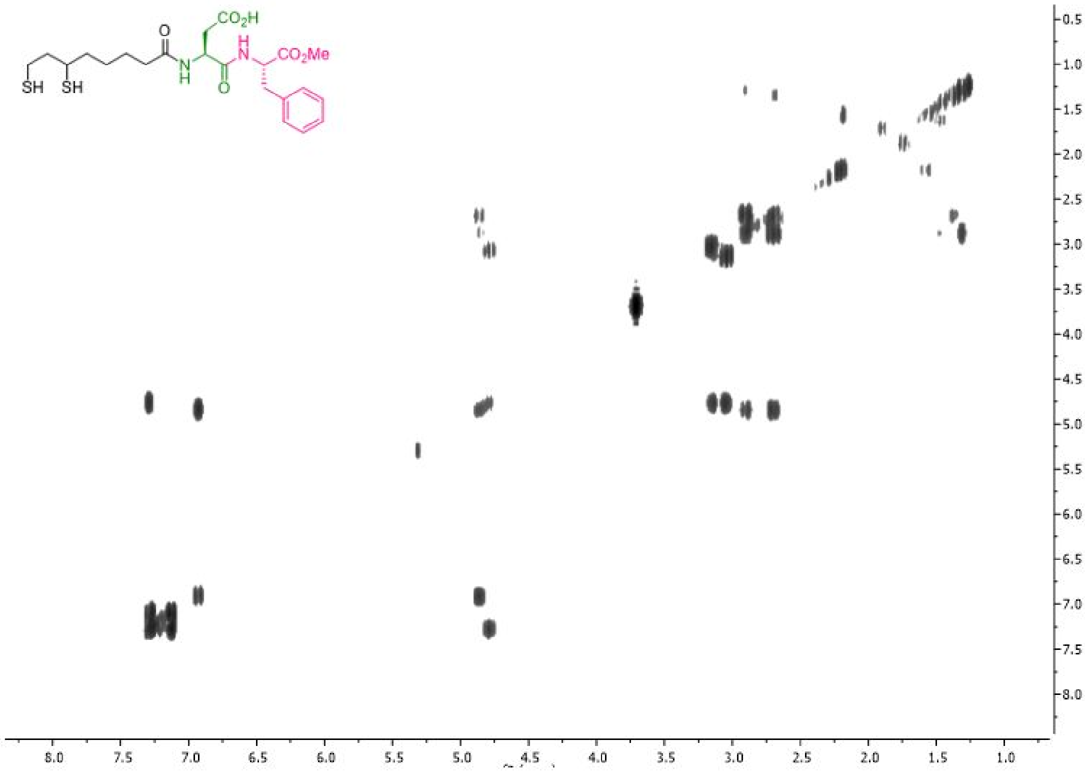

Figure S7 COSY NMR spectra of **DHLA-Asptm**

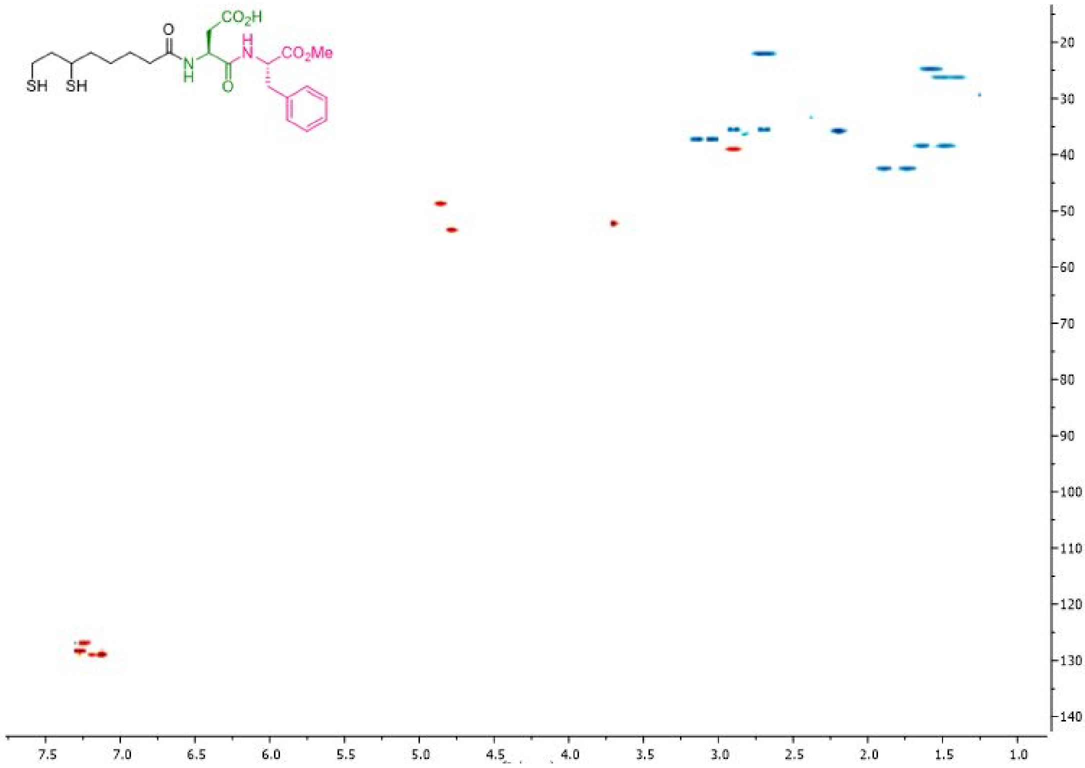

Figure S8 HSQC NMR spectra of **DHLA-Asptm**

We used Au^{3+} ions with 2 mM and 10 mM concentrations for synthesis of DHLA directed Au NPs. The mixture consisting of 2 mM Au^{3+} ions and DHLA was exposed to the same UV light, there was no Au NPs formation in first 20 min photoirradiation. Initial yellow color of the mixture was turned to brownish color in 30 min and 40 min photoirradiation (Figure S9A), which can be considered to be formation of the Au NPs. While the absorbance spectrum of the Au NP solutions in Figure S9B was consistent with Figure S9A, the Au NPs formed at 30 min (blue line) and 40 min (purple line) photoirradiation gave very broad peaks at around 650 nm, which means that the Au NPs were not uniform and it did not show good colloidal property.

When we increased the concentration of Au^{3+} ions to 10 mM, no formation of the Au NPs was observed during the 40 min photoirradiation. As seen that initial yellow color of the Au^{3+} ions mixture remained unchanged during the 40 min photoirradiation (Figure S9C) and there was no characteristic absorbance peak of Au NPs in absorbance spectrum. The Figure posted below was given in the Supporting Information (Figure S9D).

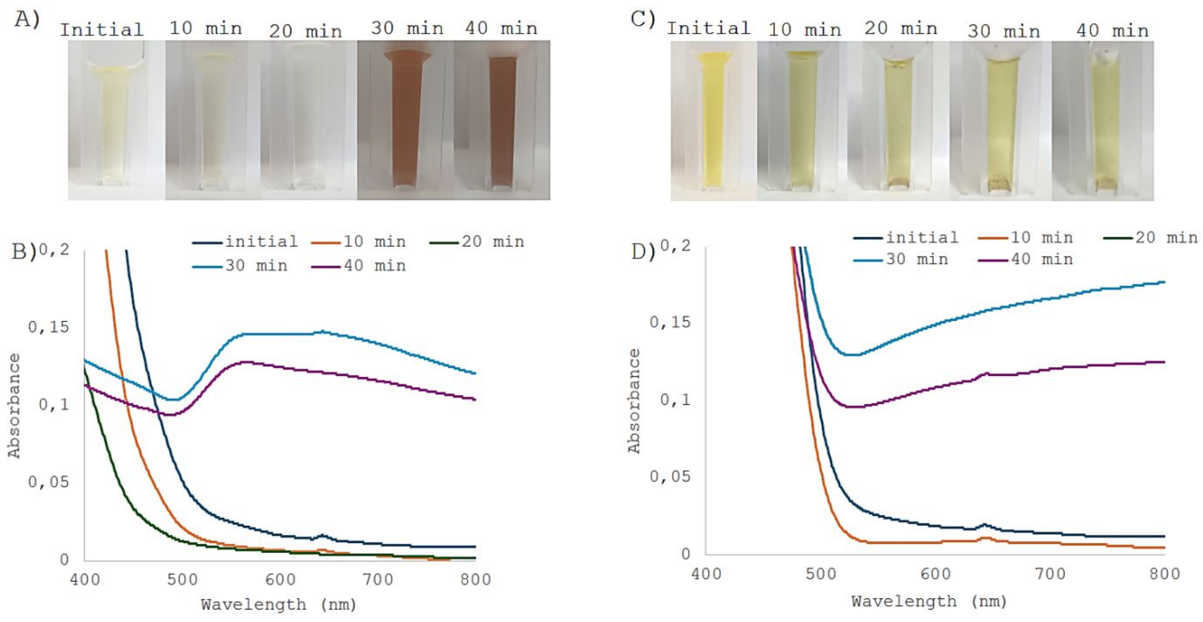

Figure S9. Synthesis of Au NPs under photoirradiation with different concentrations of Au^{3+} ions: A-B) 2 mM and C-D) 10 mM
